# Internet Addiction in Socio-Demographic, Academic, and Psychological Profile of College Students During the COVID-19 Pandemic in the Czech Republic and Slovakia

**DOI:** 10.3389/fpubh.2022.944085

**Published:** 2022-06-23

**Authors:** Beata Gavurova, Viera Ivankova, Martin Rigelsky, Tawfik Mudarri

**Affiliations:** ^1^Department of Addictology, First Faculty of Medicine, Charles University and General University Hospital in Prague, Prague, Czechia; ^2^Institute of Earth Resources, Faculty of Mining, Ecology, Process Control and Geotechnologies, Technical University of Košice, Košice, Slovakia; ^3^Department of Marketing and International Trade, Faculty of Management and Business, University of Prešov, Prešov, Slovakia

**Keywords:** mental health, problematic internet use, depressive symptoms, anxiety symptoms, stress, study-related characteristics, socio-demographic characteristics, relationships

## Abstract

Internet addiction is a serious problem among young adults that requires increased attention, especially at a time of distance learning during the coronavirus disease 2019 (COVID-19) pandemic. The aim of the study was to assess the relationships between internet addiction and selected socio-demographic, study-related, and psychological characteristics of college students. Internet addiction was measured using the Internet Addiction Test both overall and in its individual subscales (Salience, Excessive Use, Neglect Work, Anticipation, Lack of Control, and Neglect Social Life). The selected characteristics represented (1) socio-demographic profile (gender, age, residence, family), (2) academic profile (housing during the semester, form of study), and (3) psychological profile (depressive symptoms—the Patient Health Questionnaire, stress—the Perceived Stress Scale, anxiety symptoms—the Generalized Anxiety Disorder). Data collection took place during the first wave of the COVID-19 pandemic in 2020 at Czech and Slovak colleges, with 1,422 students from the Czech Republic and 1,677 students from Slovakia participating in the research. The analytical processes were carried out through descriptive analysis, non-parametric difference analysis, and multiple negative binomial regression. Mild internet addiction was found in 387 (27.2%) Czech and 452 (27.0%) Slovak students. Moderate internet addiction was identified in 49 (3.4%) students from the Czech Republic and in 100 (6.0%) students from Slovakia. Two (0.1%) Czech and three (0.2%) Slovak students reported severe internet addiction. Increased likelihood of internet addiction overall, as well as in most individual subscales, was found particularly among male students and students who lived away from home during the semester. Depressive symptoms and stress could also be considered significant predictors in both countries. These results are important for the development of effective strategies and prevention programs, as Internet addiction may be a serious problem in the future, given the current times. When assessing internet addiction among college students, it would also be appropriate to evaluate the individual internet addiction subscales and their specifics.

## Introduction

A strong attribute of today's modern times is the internet, which has become an everyday part not only of young people. The increasing accessibility of the Internet over the last two decades has significantly changed young people's lives in many dimensions. The internet is ubiquitous, whether it is for entertainment, relaxation, escaping worries, shopping, but also for seeking study information and connecting with friends and family (1, 2). However, the dark side is the fact that the internet can suck young individuals into an online world where excessive time spent online can contribute to compulsive unhealthy behavior (3). In this sense, it is possible to speak of a non-substance drug that brings with it many consequences in the social and academic spheres, but also in mental health (4, 5). In other words, the internet can help with many needs, but it can also be a harmful element if a reasonable level of use is exceeded.

Youth itself represents a complicated phase in an individual's life in which he or she may succumb to various pitfalls. Studying at college is a critical period during which young people face many challenges, which is why college students are often identified as a vulnerable group (6). Starting college is already a great change, in which the student encounters many new situations and problems. Relationships, acceptance and integration, academic performance, meeting expectations, but also future employability are important aspects at this time. The consequences of problematic internet use by college students can be poor academic performance, disinterest in engaging in other activities, excessive daytime sleepiness (7), poor sleep quality (8), reduced academic engagement (dedication and vigor) (9), risky health behaviors (10), but also health problems, such as being under- or over-weight (11, 12), migraine (12, 13), back pain (12), or increased resting heart rate (14). These are reasons for increased vigilance about young people's use of the internet. Caution is also stressed in view of the fact that college students are seen as an important future pillar of society and economic prosperity.

In addition to the facts mentioned in the previous paragraph, the coronavirus disease 2019 (COVID-19) pandemic represents another psychological burden that has also affected the lives of students (15). The measures related to COVID-19 disrupted students' education, social contact, and leisure time. This period can undoubtedly be considered psychologically challenging, as evidence shows that common mental disorders occurred frequently among students during the pandemic (16), while distance learning through digital technologies was a critical aspect for mental health (17). The regulations led to greater use of modern technology, including the internet (18, 19), which may have put young people's mental health at risk. For this reason, many studies have been conducted around the world to clarify the consequences of the COVID-19 pandemic. Compared to the pre-pandemic period, many studies showed higher rates of online activity (20–23), but also higher rates of internet addiction (24). However, it is also important to know what factors play a significant role in this problem.

Problematic internet use can be understood as using the internet in a way that has a negative influence on an individual's life and can lead to addiction (25). When a problem is identified, increased attention is focused not only on the time spent online, but especially on consequences such as loss of control, unsuccessful quit attempts, or disrupted relationships with family and friends (26). In addition, evidence clearly shows that problematic internet use is a significant predictor of depression, anxiety, and stress in young people (27, 28). On the other hand, there are also findings indicating that the presence of common mental disorders may explain problematic internet use (16). According to Masaeli and Farhadi (29), internet-based addictive behaviors during the COVID-19 pandemic were mostly due to financial hardships, isolation, problematic substance use, and psychological distress, including depression, anxiety, and stress. In this context, depression, anxiety, and stress are known to be associated with more severe problematic use and/or internet addiction in students (6, 18, 30–35). Based on these findings, it can be assumed that depressive symptoms, anxiety symptoms and perceived stress may also prove to be significant predictors in other countries, such as the Czech Republic and Slovakia. In addition, studies show that individual aspects such as gender, age, residence and academic characteristics also play an important role in problematic internet use (18, 32, 36–43).

In terms of internet addiction among college students, the pre-pandemic period is not sufficiently mapped in the Czech Republic and Slovakia, which is indicative of the fact that little attention has been paid to this issue in these two neighboring countries. For this reason, research-based knowledge and its translation into practice are lacking. In Slovakia, Sebena et al. (44) highlighted the seriousness of problematic internet use among college students and confirmed that depressive symptoms predict their problematic internet use. In the Czech Republic, the issue was addressed by Chraska (45). In both countries, however, the research focus was mainly on primary and secondary school students (46–48). Thus, to the best of the authors' knowledge, there is a lack of research on the issue among college students in both the Czech Republic and Slovakia, which can be identified as a relevant research gap. On this basis, this study examines the relationships between internet addiction and selected socio-demographic, academic, and psychological characteristics of Czech and Slovak college students during the COVID-19 pandemic. Knowledge of these relationships provides the opportunity to put in place effective protective and preventive measures to address the problem and avoid serious consequences in the future.

## Materials and Methods

The aim of the study was to assess the relationships between internet addiction and selected socio-demographic, academic, and psychological characteristics of Czech and Slovak college students. The research examined internet addiction from an overall perspective but also in sub-scales, namely Salience, Excessive Use, Neglect Work, Anticipation, Lack of Control, and Neglect Social Life. As indicated, the selected characteristics were concentrated in three areas, namely (1) socio-demographic profile (gender, age, residence, and family), (2) academic profile (housing during the semester, form of study), and (3) psychological profile (depressive symptoms, stress, and anxiety symptoms). The research was conducted based on the following research questions (RQs).

RQ1: How are selected socio-demographic characteristics associated with internet addiction in Czech and Slovak students?
RQ2: How are selected academic characteristics associated with internet addiction in Czech and Slovak students?
RQ3: How are selected psychological characteristics associated with internet addiction in Czech and Slovak students?

### Measures

In addition to the usual socio-demographic and academic identification data, the online questionnaire also collected data on students' level of psychological distress.

Psychological distress was measured using already existing scales from previous studies. Thus, depressive symptoms were measured using the Patient Health Questionnaire (PHQ-9) and anxiety symptoms using the Generalized Anxiety Disorder (GAD-7) screening scale from the study by Kroenke et al. (49). The PHQ-9 has been widely validated to screen for depressive symptoms (50) and is considered a suitable instrument also for a sample of college students (51, 52). Similarly, the GAD-7 is a commonly used instrument for screening anxiety symptoms among college students and show good psychometric properties (53–55). For these scales, respondents could choose one of the following answers for each questionnaire item: not at all-−0, several days-−1, more than half the days-−2, nearly every day-−3. The total scores were the sum of the coded responses. Based on this, depressive symptoms were identified as follows: no depressive symptoms (0–4), mild depressive symptoms (5–9), moderate depressive symptoms (10–14), moderately severe depressive symptoms (15–19), and severe depressive symptoms (20 or more). Anxiety symptoms were identified as follows: no anxiety symptoms (0–4), mild anxiety symptoms (5–9), moderate anxiety symptoms (10–14), and severe anxiety symptoms (15 or more).

Perceived stress was measured using the Perceived Stress Scale (PSS-10) scale from the study by Cohen et al. (56). This instrument, with its many benefits, is widely recommended for use across cultures and also in student populations (57–59). In this case, respondents could select one of the following answers for each of the 10 items: never-−0, almost never-−1, sometimes-−2, fairly often-−3, very often-−4. The ranges of the total PSS-10 score indicated: low stress (0–13), moderate stress (14–26), and high stress (27–40). Accordingly, the higher the total score, the more severe the psychological distress.

The research focused predominantly on internet addiction as measured by the Internet Addiction Test (IAT) developed by Young (60). The IAT represents an instrument with satisfactory psychometric properties that is commonly used in studies focusing on college students (61, 62). The scale consisted of 20 items with the following answers: not applicable-−0, rarely-−1, occasionally-−2, frequently-−3, often-−4, always-−5. Internet addiction was identified as follows: normal level of internet usage (0–30), mild addiction symptoms (31–49), moderate addiction symptoms (50–79), and severe addiction symptoms (80–100). The IAT scale was composed of six subscales for which precise interval thresholds are not defined. Therefore, higher score (closer to the theoretical maximum) represented greater addiction. Young (63) explained these subscales as follows:

- Salience. A high score on salience-related items points to the fact that the individual is likely to feel preoccupied with the internet, hides his/her behavior from others, and may show a loss of interest in other activities and/or relationships due to a preference for more solitary time on the internet. A high score also shows that the individual uses the internet as a form of mental escape from disturbing thoughts and may feel that life without the internet would be boring, empty, and/or joyless. Salience is represented by five items (ID: 10, 12, 13, 15, 19) with a theoretical score range of 0–25.
- Excessive Use. A high score on items related to excessive use reflects the fact that the individual engages in excessive online behavior and compulsive usage, and is intermittently unable to control his or her time spent on the internet, which he or she hides from others. The individual with a high score is very likely to fall into depression, panic, or anger if forced to stay off the internet for a prolonged period of time. Excessive use is represented by five items (ID: 1, 2, 14, 18, 20) with a theoretical score range of 0–25.
- Neglect Work. A high score on items related to neglect work points to the fact that the individual may consider the internet to be a necessary appliance, much like a television or telephone. Given the amount of time spent online, work or school performance and productivity are likely to deteriorate, and the individual may begin to defend or hide time spent online. Time spent on the internet conflicts with responsibilities (duties, school, and work). Neglect work is represented by three items (ID: 6, 8, 9) with a theoretical score range of 0–15.
- Anticipation. A high score on anticipation-related items shows that the individual is most likely to think about being online when not at a computer, and feel compelled to use the internet when offline. Thus, it is a compulsion to be on the internet. Anticipation is represented by two items (ID: 7, 11) with a theoretical score range of 0–10.
- Lack of Control. A high score on items related to lack of control items indicates that the individual has difficulty managing his or her time online. The individual often stays online longer than intended, and others may complain about the amount of time he or she spends online. Lack of control is represented by three items (ID: 5, 16, 17) with a theoretical score range of 0–15.
- Neglect Social Life. A high score on items related to neglecting social life points to the fact that the individual is likely to use online relationships to cope with situational problems and/or to reduce psychological strain and stress. The individual often establishes new relationships with other internet users and uses the internet to make social contacts that may be lacking in his/her life. Thus, it is about replacing offline social life with online. Neglect social life is represented by two items (ID: 3, 4) with a theoretical score range of 0–10.

Table 1 shows the IAT scale items, with the ID column representing the item number for easier assignment to individual subscales.

**Table 1:** IAT scale items.

**ID**	**Item**	**CZ**			**SK**		
		**Med**	**Mean**	**SD**	**Med**	**Mean**	**SD**
1	How often do you find that you stay online longer than you intended?	3.00	2.53	1.221	3.00	2.71	1.223
2	How often do you neglect household chores to spend more time online?	2.00	1.69	1.092	2.00	1.71	1.153
3	How often do you prefer the excitement of the internet to intimacy with your partner?	0.00	0.56	0.906	0.00	0.49	0.894
4	How often do you form new relationships with fellow online users?	1.00	1.10	1.000	1.00	1.10	0.988
5	How often do others in your life complain to you about the amount of time you spend online?	1.00	1.16	1.049	1.00	1.19	1.080
6	How often do your grades or school work suffer because of the amount of time you spend online?	2.00	1.97	1.225	2.00	1.89	1.256
7	How often do you check your email before something else that you need to do?	3.00	2.51	1.395	2.00	2.48	1.366
8	How often does your job performance or productivity suffer because of the internet?	2.00	2.16	1.210	2.00	2.03	1.210
9	How often do you become defensive or secretive when anyone asks you what you do online?	1.00	1.02	1.095	1.00	1.05	1.194
10	How often do you block out disturbing thoughts about your life with soothing thoughts of the internet?	1.00	1.19	1.244	1.00	1.20	1.282
11	How often do you find yourself anticipating when you will go online again?	1.00	0.78	0.912	1.00	0.86	1.053
12	How often do you fear that life without the internet would be boring, empty, and joyless?	1.00	0.95	1.081	1.00	0.96	1.147
13	How often do you snap, yell, or act annoyed if someone bothers you while you are online?	1.00	0.83	0.978	1.00	1.00	1.067
14	How often do you lose sleep as a result of late-night log-ins?	1.00	1.19	1.209	1.00	1.31	1.317
15	How often do you feel preoccupied about the internet when offline, or fantasize about being online?	0.00	0.68	0.886	0.00	0.67	0.928
16	How often do you find yourself saying “just a few more minutes” when online?	1.00	1.51	1.351	1.00	1.70	1.415
17	How often do you try to cut down the amount of time you spend online and fail?	1.00	1.35	1.190	1.00	1.60	1.270
18	How often do you try to hide how long you've been online?	0.00	0.65	0.957	0.00	0.74	1.003
19	How often do you choose to spend more time online over going out with others?	1.00	0.86	1.043	1.00	0.86	1.104
20	How often do you feel depressed, moody, or nervous when you are offline, a feeling which goes away once you are back online?	0.00	0.42	0.789	0.00	0.45	0.782

*SD, standard deviation; CZ, Czech Republic; SK, Slovakia*.

### Data Collection Process and Sample Selection

Respondents completed an online questionnaire distributed in the first half of 2020 (the first wave of the COVID-19 pandemic in the Czech Republic and Slovakia). Each respondent was given the same information and instructions and no one was offered a reward. Respondents completed the questionnaires in their national language, i.e., Czech or Slovak. The translation was done from English into Slovak and then from Slovak into Czech. The Slovak version was chosen for translation into Czech on the basis of the high similarity between the Slovak and Czech languages. The translated versions were verified by experts and tested on a group of students to confirm their understanding of the questionnaire items.

The data collection process was carried out in two steps. In the first step, representatives of colleges (deans, vice-deans, study officers) and student councils were asked by emails to share the online questionnaire among their students and student groups, and invited them to complete it. The questionnaire was also distributed to students through the social network Facebook. In the second step, addressed emails (from publicly available contact databases) were sent to college teachers of certain fields of study in some colleges requesting them to distribute the questionnaire to their students. The aim of the second step was to reach the missing segments of students in order to achieve the target sample structure. In order to have a representative sample in line with the study population, the sample was monitored based on the criterion of representation of colleges. In both countries, 80% of all colleges were covered. The interest was also to obtain a sample with a more consistent proportion of study fields and to have at least 30 observations for each field.

**Figure 1:**
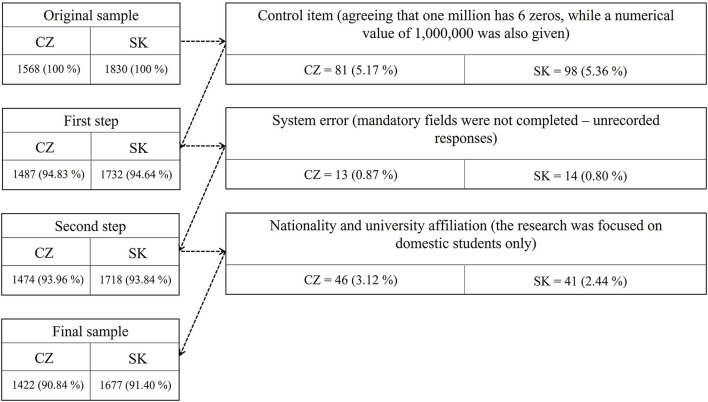
Selection process to obtain the final sample.

With regard to data processing, procedures were carried out to exclude irrelevant statistical units. Thus, 179 statistical units were excluded on the basis of a control item, 27 statistical units on the basis of the identification of a system error in the recording of responses, and 87 statistical units on the basis of the foreign nationality of the students (the survey focused on domestic students). In total, the research sample included 3,099 respondents (Czech Republic = 1,422; Slovakia = 1,677). For some identifying characteristics, respondents provided an apparently incorrect answer (e.g., 1,000 as the year of birth), these responses were deleted and appear as missing data in the analyzes. The selection process to obtain the final sample is provided in Figure 1.

Table 2 shows the basic characteristics of the research sample. Although the Czech Republic and Slovakia share a common history, the current specificities of these countries mean that each country should be evaluated separately. On this basis, it was possible to identify some inconsistencies. In terms of gender, higher proportions of female students were identified. This phenomenon is the result of a higher willingness of females to answer questionnaires, but also of the fact that more females (60%) than males (40%) attend college in the selected countries (64). The increased proportions in the younger age groups can be explained by the fact that students tend to be at a young age and older students (mostly part-time) represent a disproportionately smaller group in the higher education environment.

**Table 2:** Profile of the sample.

**Variable**	**CZ**			**SK**		
	***n***	**%**	**% without missing**	***n***	**%**	**% without missing**
**Gender**						
Male	349	24.5	24.5	606	36.1	36.1
Female	1,073	75.5	75.5	1,071	63.9	63.9
**Age**						
≤ 20	193	13.6	13.6	206	12.3	12.3
21–25	891	62.7	62.7	1,239	73.9	74.1
26–30	171	12	12	143	8.5	8.5
≥31	166	11.7	11.7	85	5.1	5.1
Missing	1	0.1	-	4	0.2	-
**Place of Residence**						
Rural	457	32.1	32.1	823	49.1	49.1
Urban	965	67.9	67.9	854	50.9	50.9
**Family**						
Complete	1,020	71.7	71.7	1,359	81	81
Incomplete	402	28.3	28.3	318	19	19
**Housing during the semester**						
Away from home	772	54.3	54.3	939	56	56
Home	650	45.7	45.7	738	44	44
**Form of study**						
Full-time	1,041	73.2	73.2	1,550	92.4	92.4
Part-time	381	26.8	26.8	127	7.6	7.6
**PHQ-9**						
No	683	48	48	877	52.3	52.3
Mild	406	28.6	28.6	479	28.6	28.6
Moderate	193	13.6	13.6	185	11	11
Moderately severe	91	6.4	6.4	90	5.4	5.4
Severe	49	3.4	3.4	46	2.7	2.7
**PSS-10**						
Low	186	13.1	13.1	193	11.5	11.5
Moderate	1,053	74.1	74.1	1,331	79.4	79.4
High	183	12.9	12.9	153	9.1	9.1
**GAD-7**						
No	849	59.7	59.7	1,097	65.4	65.4
Mild	372	26.2	26.2	385	23	23
Moderate	131	9.2	9.2	136	8.1	8.1
Severe	70	4.9	4.9	59	3.5	3.5
**Field of study**						
Education	277	19.5	19.5	80	4.8	4.8
Humanities and Arts	101	7.1	7.1	78	4.7	4.7
Social, Economic and Legal Sciences	665	46.8	46.8	671	40	40
Natural Science	50	3.5	3.5	73	4.4	4.4
Design, Technology, Production and Communications	93	6.5	6.5	164	9.8	9.8
Agricultural and Veterinary Sciences	67	4.7	4.7	53	3.2	3.2
Health Service	54	3.8	3.8	180	10.7	10.7
Services (tourism, sports, security, transport, logistics)	69	4.9	4.9	240	14.3	14.3
Informatics, Mathematics & ICT	46	3.2	3.2	138	8.2	8.2

*CZ, Czech Republic; SK, Slovakia; n, frequency; ICT, Information and Communication Technologies*.

### Statistical Analysis

The analysis focused on describing the data along with testing for differences, and on assessing possible relationships. Non-parametric tests of differences were used to assess differences, namely the Mann Whitney *U*-test for two categories and the Kruskal-Wallis *H*-test for three or more categories. Multiple negative binomial regression was used to assess the relationships, where the dependent variable was IAT and its subscales and the independent variables were characteristics such as gender, age, residence (urban/rural), family (incomplete/complete), housing during the semester (at home/away from home), form of study (part-time/full-time), PHQ-9, PSS-10, and GAD-7. The reference categories for nominal variables were selected with an emphasis on the frequency of observations in a given category and the logical interpretation of the results.

Statistical processing was performed using SPSS Statistic v. 26 (IBM, Inc., Armonk, NY, US) and programming language R v 4.1.2 (65).

## Results

This section presents the results of the analytical procedures. First, the mean values of the total IAT scores and the IAT subscale scores in the sorting categories of the selected characteristics are presented. The presentation of results is provided in order of gender, age, residence, family, housing during the semester, form of study, depressive symptoms (PHQ-9), stress (PSS-10), and anxiety symptoms (GAD-7). Subsequently, the results of a regression model focusing on the relationships between Internet addiction and these selected characteristics are presented.

### Results of Frequency Analysis

In terms of overall IAT scores, 984 (69.2%) Czech students and 1,122 (66.9%) Slovak students showed no symptoms of internet addiction, i.e., normal internet usage. Mild internet addiction was found in 387 (27.2%) Czech and 452 (27.0%) Slovak students. Moderate internet addiction was identified in 49 (3.4%) students from the Czech Republic and in 100 (6.0%) students from Slovakia. Two (0.1%) Czech and three (0.2%) Slovak students reported severe internet addiction.

### Results of Descriptive and Difference Analyzes

In Tables 3–**11**, the IAT subscales were compared between groups of students with selected sociodemographic, academic, and psychological characteristics. Socio-demographic and academic characteristics make it possible to identify groups of students who should be given increased attention. Psychological characteristics make it possible to capture the intensity of mental health problems that may co-occur with increased internet addiction.

**Table 3:** Mean values of the IAT and its subscales in the classification by gender.

**Country**	**Gender**	**Salience**	**Excessive use**	**Neglect work**	**Anticipation**	**Lack of control**	**Neglect social life**	**IAT**
CZ	Male	4.97	6.24	5.40	3.09	4.42	2.23	25.69
	Female	4.37	6.57	5.07	3.36	4.50	1.48	24.95
	Diff^*^ sig. α <0.05	Yes	No	No	Yes	No	Yes	No
SK	Male	5.50	7.39	5.34	3.37	4.88	1.92	28.08
	Female	4.24	6.66	4.76	3.32	4.56	1.40	24.84
	Diff^*^ sig. α <0.05	Yes	Yes	Yes	No	Yes	Yes	Yes

Diff^*^ sig. α < 0.05—difference at a significance level of α < 0.05 using Mann-Whitney U-test; CZ, Czech Republic; SK, Slovakia; IAT, Internet Addiction Test.

Based on the results from Table 3, it was possible to confirm gender differences in Salience, Anticipation and Neglect Social Life in the Czech Republic. In the case of Salience and Neglect Social Life, higher mean values were measured for males, while in the case of Anticipation, a higher value was found for females. There was no difference in the total IAT scores between Czech male and female students. In contrast, significant gender differences between Slovak students were evident in all cases except Anticipation. In these significant cases, higher mean values were measured for males.

Table 4 shows the results of testing the differences in the IAT and its subscales between students in the age classification. Significant differences were evident among both Czech and Slovak students. The only case in which no significant difference was found was Neglect Social Life in the Slovak sample. With a focus on mean values, the most pronounced difference was observed in the oldest age group (31+), where the lowest values were found.

**Table 4:** Mean values of the IAT and its subscales in the classification by age.

**Country**	**Age**	**Salience**	**Excessive use**	**Neglect work**	**Anticipation**	**Lack of control**	**Neglect social life**	**IAT**
CZ	≤ 20	4.78	6.78	5.63	3.46	4.65	1.56	26.83
	21–25	4.74	6.82	5.44	3.34	4.88	1.71	26.35
	26–30	4.36	5.85	4.78	3.10	3.82	1.78	23.13
	≥31	3.13	5.04	3.44	3.03	2.86	1.43	18.67
	Diff^*^ sig. α <0.05	Yes	Yes	Yes	Yes	Yes	Yes	Yes
SK	≤ 20	5.08	7.24	5.34	3.41	4.95	1.65	27.71
	21–25	4.72	6.94	5.01	3.30	4.72	1.57	26.01
	26–30	4.79	7.30	5.06	3.87	4.56	1.72	27.21
	≥31	3.41	5.24	3.28	2.94	3.47	1.54	19.66
	Diff^*^ sig. α <0.05	Yes	Yes	Yes	Yes	Yes	No	Yes

Diff^*^ sig. α < 0.05—difference at a significance level of α < 0.05 using Mann-Whitney U-test; CZ, Czech Republic; SK, Slovakia; IAT, Internet Addiction Test.

The results from Table 5 indicated that whether students lived in rural or urban areas was unlikely to have a decisive effect in the development of Internet addiction. This was apparent from the fact that the only significant difference between urban and rural students was found in Lack of Control. In this analyzed case, a higher mean value was measured for Czech rural students.

**Table 5:** Mean values of the IAT and its subscales in the classification by residence.

**Country**	**Residence**	**Salience**	**Excessive use**	**Neglect work**	**Anticipation**	**Lack of control**	**Neglect social life**	**IAT**
CZ	Rural	4.55	6.43	5.28	3.33	4.71	1.54	25.38
	Urban	4.50	6.52	5.09	3.27	4.37	1.72	25.02
	Diff^*^ sig. α <0.05	No	No	No	No	Yes	No	No
SK	Rural	4.49	6.71	4.96	3.37	4.72	1.54	25.59
	Urban	4.90	7.13	4.98	3.31	4.64	1.64	26.41
	Diff^*^ sig. α <0.05	No	No	No	No	No	No	No

Diff^*^ sig. α < 0.05—difference at a significance level of α < 0.05 using Mann-Whitney U-test; CZ, Czech Republic; SK, Slovakia; IAT, Internet Addiction Test.

Similar to residence, the family in which college students grew up did not appear to be significant in the development of internet addiction. This was supported by the results in Table 6, where no significant difference was found.

**Table 6:** Mean values of the IAT and its subscales in the classification by family.

**Country**	**Family**	**Salience**	**Excessive use**	**Neglect work**	**Anticipation**	**Lack of control**	**Neglect social life**	**IAT**
CZ	Complete	4.49	6.54	5.21	3.32	4.51	1.64	25.27
	Incomplete	4.58	6.35	5.00	3.23	4.41	1.71	24.79
	Diff^*^ sig. α <0.05	No	No	No	No	No	No	No
SK	Complete	4.70	6.85	4.96	3.36	4.65	1.58	25.93
	Incomplete	4.69	7.24	5.01	3.26	4.80	1.62	26.35
	Diff^*^ sig. α <0.05	No	No	No	No	No	No	No

Diff^*^ sig. α < 0.05—difference at a significance level of α < 0.05 using Mann-Whitney U-test; CZ, Czech Republic; SK, Slovakia; IAT, Internet Addiction Test.

In addition to the mean values, Table 7 shows the results of testing the differences in the IAT and its subscales between students who lived at home during the semester and students who lived away from home during the semester (e.g., dormitory, private accommodation, etc.). In the Czech sample, significant differences were identified in all cases except Anticipation. In the Slovak sample, significant differences were not found in two cases, namely Anticipation and Neglect Social Life. In all significant cases, higher mean values of internet addiction and its subscales were measured for students who lived away from home during the semester.

**Table 7:** Mean values of the IAT and its subscales in the classification by housing during the semester.

**Country**	**Housing during the semester**	**Salience**	**Excessive use**	**Neglect work**	**Anticipation**	**Lack of control**	**Neglect social life**	**IAT**
CZ	Away from home	4.96	7.01	5.49	3.34	4.69	1.75	26.84
	Home	4.00	5.87	4.74	3.24	4.23	1.56	23.11
	Diff^*^ sig. α <0.05	Yes	Yes	Yes	No	Yes	Yes	Yes
SK	Away from home	5.02	7.35	5.37	3.41	4.99	1.64	27.58
	Home	4.28	6.38	4.46	3.25	4.28	1.53	24.01
	Diff^*^ sig. α <0.05	Yes	Yes	Yes	No	Yes	No	Yes

Diff^^*^^ sig. α < 0.05—difference at a significance level of α < 0.05 using Mann-Whitney U-test; CZ, Czech Republic; SK, Slovakia; IAT, Internet Addiction Test.

Significant differences were also revealed in the study form, which are presented along with the mean values in Table 8. For both Czech and Slovak students, significant differences were consistently observed in the total IAT score as well as in all its subscales except Anticipation and Neglect Social Life. Accordingly, Czech and Slovak full-time students reported higher mean values than part-time students.

**Table 8:** Mean values of the IAT and its subscales in the classification by form of study.

**Country**	**Form of study**	**Salience**	**Excessive use**	**Neglect work**	**Anticipation**	**Lack of control**	**Neglect social life**	**IAT**
CZ	Full-time	4.75	6.77	5.46	3.34	4.76	1.68	26.28
	Part-time	3.87	5.72	4.30	3.17	3.72	1.62	22.00
	Diff^*^ sig. α <0.05	Yes	Yes	Yes	No	Yes	No	Yes
SK	Full-time	4.77	7.01	5.06	3.35	4.77	1.59	26.36
	Part-time	3.84	5.93	3.90	3.25	3.59	1.54	21.74
	Diff^*^ sig. α <0.05	Yes	Yes	Yes	No	Yes	No	Yes

Diff^*^ sig. α < 0.05—difference at a significance level of α < 0.05 using Mann-Whitney U-test; CZ, Czech Republic; SK, Slovakia; IAT, Internet Addiction Test.

Table 9 presents the mean values and the results of testing for differences between students with different severity of depressive symptoms. In all cases, significant differences were confirmed, with a higher severity of depression being associated with a higher level of internet addiction. This was indicated by the mean values, which increased with higher severity of depressive symptoms in most cases. In other words, Czech and Slovak students with more severe depressive symptoms also showed higher scores for internet addiction and its subscales.

**Table 9:** Mean values of the IAT and its subscales in the classification by severity of depressive symptoms.

**Country**	**PHQ-9**	**Salience**	**Excessive use**	**Neglect work**	**Anticipation**	**Lack of control**	**Neglect social life**	**IAT**
CZ	No	3.45	5.25	4.37	2.97	3.72	1.53	20.80
	Mild	4.88	6.99	5.47	3.36	4.79	1.57	26.58
	Moderate	5.96	7.69	6.02	3.69	5.38	1.99	30.31
	Moderately severe	6.33	9.35	6.57	4.01	5.91	2.14	33.88
	Severe	7.31	9.59	7.39	4.31	6.33	2.12	36.92
	Diff^*^ sig. α <0.05	Yes	Yes	Yes	Yes	Yes	Yes	Yes
SK	No	3.61	5.74	4.07	3.00	3.88	1.42	21.48
	Mild	5.14	7.42	5.53	3.57	5.09	1.67	28.28
	Moderate	6.77	8.76	6.41	4.07	6.04	1.88	33.66
	Moderately severe	6.93	9.44	6.57	3.56	6.17	1.81	34.68
	Severe	8.11	11.91	7.54	4.22	7.24	2.35	41.07
	Diff^*^ sig. α <0.05	Yes	Yes	Yes	Yes	Yes	Yes	Yes

Diff^*^ sig. α < 0.05—difference at a significance level of α < 0.05 using Mann-Whitney U-test; CZ, Czech Republic; SK, Slovakia; PHQ-9, Patient Health Questionnaire for depressive symptoms; IAT, Internet Addiction Test.

From Table 10, it was possible to conclude that the higher mean values of the total IAT scores, as well as the scores in the vast majority of the IAT subscales, were concentrated in the higher levels of stress. Thus, Czech and Slovak students with higher stress also reported higher internet addiction. Significant differences were found in most of the analyzed cases, with the exception of only one case, namely Neglect Social Life in the Czech sample.

**Table 10:** Mean values of the IAT and its subscales in the classification by stress level.

**Country**	**PSS-10**	**Salience**	**Excessive use**	**Neglect work**	**Anticipation**	**Lack of control**	**Neglect social life**	**IAT**
CZ	Low	2.99	4.79	4.01	2.76	3.31	1.68	18.92
	Moderate	4.46	6.42	5.12	3.26	4.45	1.64	24.89
	High	6.39	8.59	6.51	4.01	5.87	1.78	32.82
	Diff^*^ sig. α <0.05	Yes	Yes	Yes	Yes	Yes	No	Yes
SK	Low	2.75	5.00	3.51	2.76	3.35	1.28	18.40
	Moderate	4.73	6.89	4.98	3.39	4.68	1.61	26.09
	High	6.90	9.65	6.78	3.68	6.31	1.82	34.93
	Diff^*^ sig. α <0.05	Yes	Yes	Yes	Yes	Yes	Yes	Yes

Diff^*^ sig. α < 0.05—difference at a significance level of α < 0.05 using Mann-Whitney U-test; CZ, Czech Republic; SK, Slovakia; PSS-10, Perceived Stress Scale; IAT, Internet Addiction Test.

The results in Table 11 point to the fact that higher mean scores for internet addiction were observed in Czech and Slovak students with more severe anxiety symptoms. In all cases, significant differences in internet addiction were found between groups of students with different severity of anxiety symptoms.

**Table 11:** Mean values of the IAT and its subscales in the classification by severity of anxiety symptoms.

**Country**	**GAD-7**	**Salience**	**Excessive use**	**Neglect work**	**Anticipation**	**Lack of control**	**Neglect social life**	**IAT**
CZ	No	3.85	5.75	4.75	3.06	4.05	1.57	22.54
	Mild	5.09	7.19	5.44	3.45	4.93	1.73	27.35
	Moderate	6.19	8.01	6.08	3.93	5.31	1.80	30.91
	Severe	6.39	8.90	6.76	4.06	5.73	2.14	33.93
	Diff^*^ sig. α <0.05	Yes	Yes	Yes	Yes	Yes	Yes	Yes
SK	No	4.10	6.26	4.57	3.15	4.29	1.51	23.66
	Mild	5.44	7.65	5.50	3.63	5.14	1.66	28.83
	Moderate	6.55	8.72	6.14	3.83	5.80	1.82	32.87
	Severe	6.68	10.34	6.39	3.88	6.34	2.00	35.46
	Diff^*^ sig. α <0.05	Yes	Yes	Yes	Yes	Yes	Yes	Yes

Diff^*^ sig. α < 0.05—difference at a significance level of α < 0.05 using Mann-Whitney U-test; CZ, Czech Republic; SK, Slovakia; GAD-7, Generalized Anxiety Disorder instrument; IAT, Internet Addiction Test.

### Results of Multiple Negative Binomial Regression Analysis

The multiple negative binomial regression was used to assess the relationships between selected socio-demographic, academic, and psychological characteristics of Czech and Slovak college students and internet addiction. In this analysis, several variables (compared to previous outputs) appeared as cardinal variables. These variables were age (CZ = mean: 24.8 ± 6.2, min: 19, max: 52; SK = mean: 23.5 ± 4.5, min: 18, max: 54), PHQ-9 (CZ = mean: 6.3 ± 5.5, min: 0, max: 27; SK = mean: 5.8 ± 5.3, min: 0 max: 27), PSS-10 (CZ = mean: 19.8 ± 5.6, min: 4, max: 38; SK = mean: 19.3 ± 5.3, min: 2, max: 39), and GAD-7 (CZ = mean: 4.7 ± 4.6, min: 0, max: 21; SK = mean: 4.2 ± 4.3, min: 0, max: 21).

Table 12 presents the results of the analysis focusing on the possible relationships between the analyzed variables, and this table is divided into two parts by country—Slovakia (SK) and the Czech Republic (CZ). The table provides information on the β coefficient (positive coefficients present positive relationships and vice versa), *p*-value (relationships significant at α < 0.05 are highlighted), standard error, and odds ratio (values >1 present positive relationships). As can be seen, a significant number of the analyzed relationships proved to be significant.

**Table 12:** Relationships of the IAT and its subscales with selected socio-demographic, academic, and psychological characteristics.

**Negative binomial regression**	**Salience**	**Excessive use**	**Neglect work**	**Anticipation**	**Lack of control**	**Neglect social life**	**IAT total**
	***β p*-value (SE) OR**	***β p*-value (SE) OR**	***β p*-value (SE) OR**	***β p*-value (SE) OR**	***β p*-value (SE) OR**	***β p*-value (SE) OR**	***β p*-value (SE) OR**
**SK**							
α	**0.608**^†^ **(0.161) 1.84**	**1.433**^†^ **(0.103) 4.19**	**1.269**^†^ **(0.109) 3.56**	**0.947**^†^ **(0.107) 2.58**	**1.131**^†^ **(0.116) 3.1**	−0.042 (0.168) 0.96	**2.728**^†^ **(0.096) 15.3**
Gender: Male (ref.: Female)	**0.301**^†^ **(0.041) 1.35**	**0.13**^†^ **(0.026) 1.14**	**0.148**^†^ **(0.027) 1.16**	0.028 (0.028) 1.03	**0.101**^†^ **(0.029) 1.11**	**0.338**^†^ **(0.043) 1.4**	**0.157**^†^ **(0.025) 1.17**
Age	−0.003 (0.006) 1	−0.006 (0.004) 0.99	−0.011^***^(0.004) 0.99	0.002 (0.004) 1	−0.005 (0.004) 0.99	<0.001 (0.006) 1	−0.005 (0.003) 1
Residence: Urban (ref.: Rural)	0.064 (0.04) 1.07	0.052^**^(0.026) 1.05	<0.001 (0.026) 1	−0.018 (0.027) 0.98	−0.021 (0.028) 0.98	0.032 (0.043) 1.03	0.021 (0.024) 1.02
Family: Incomplete (ref.: Complete)	−0.07 (0.051) 0.93	0.017 (0.032) 1.02	−0.012 (0.033) 0.99	−0.036 (0.035) 0.96	0.013 (0.036) 1.01	0.002 (0.053) 1	−0.022 (0.031) 0.98
Housing during the semester: Home (ref.: Away from home)	−0.112^***^(0.041) 0.89	**−0.105**^†^ **(0.026) 0.9**	**−0.127**^†^ **(0.027) 0.88**	−0.032 (0.028) 0.97	**−0.1**^†^ **(0.029) 0.9**	−0.053 (0.043) 0.95	**−0.096**^†^ **(0.025) 0.91**
Form of study: Part-time (ref.: Full-time)	−0.101 (0.095) 0.9	−0.04 (0.061) 0.96	−0.073 (0.064) 0.93	−0.013 (0.064) 0.99	−0.155^**^(0.07) 0.86	−0.002 (0.099) 1	−0.078 (0.056) 0.93
PHQ-9	**0.207**^†^ **(0.027) 1.23**	**0.154**^†^ **(0.017) 1.17**	**0.169**^†^ **(0.017) 1.18**	**0.084**^†^ **(0.018) 1.09**	**0.161**^†^ **(0.018) 1.17**	**0.109**^†^ **(0.028) 1.11**	**0.164**^†^ **(0.016) 1.18**
PSS-10	**0.293**^†^ **(0.051) 1.34**	**0.166**^†^ **(0.033) 1.18**	**0.193**^†^ **(0.033) 1.21**	0.043 (0.035) 1.04	**0.169**^†^ **(0.036) 1.18**	0.116^**^(0.053) 1.12	**0.185**^†^ **(0.031) 1.2**
GAD-7	−0.04 (0.035) 0.96	−0.014 (0.022) 0.99	−0.073^***^(0.023) 0.93	0.001 (0.024) 1	−0.049^**^(0.024) 0.95	−0.031 (0.037) 0.97	−0.036^*^ (0.021) 0.97
Pseudo *R*^2^ - Nagelkerke	0.15	0.181	0.183	0.036	0.139	0.062	0.194
**CZ**							
α	**1.129**^†^ **(0.146) 3.09**	**1.581**^†^ **(0.097) 4.86**	**1.727**^†^ **(0.094) 5.62**	**1.059**^†^ **(0.103) 2.88**	**1.61**^†^ **(0.106) 5**	0.259^*^ (0.153) 1.3	**3.06**^†^ **(0.087) 21.32**
Gender: Male (ref.: Female)	**0.226**^†^ **(0.049) 1.25**	0.012 (0.033) 1.01	**0.116**^†^ **(0.03) 1.12**	−0.033 (0.036) 0.97	0.039 (0.035) 1.04	**0.451**^†^ **(0.049) 1.57**	0.093^***^(0.03) 1.1
Age	**−0.015**^†^ **(0.005) 0.98**	−0.007^**^(0.003) 0.99	**−0.02**^†^ **(0.003) 0.98**	−0.007^**^(0.003) 0.99	**−0.022**^†^ **(0.003) 0.98**	−0.005 (0.005) 1	**−0.013**^†^ **(0.003) 0.99**
Residence: Urban (ref.: Rural)	−0.017 (0.044) 0.98	0.018 (0.029) 1.02	−0.028 (0.028) 0.97	−0.016 (0.031) 0.98	−0.071^**^(0.031) 0.93	0.089^*^ (0.048) 1.09	−0.012 (0.027) 0.99
Family: Incomplete (ref.: Complete)	−0.007 (0.046) 0.99	−0.059^*^ (0.031) 0.94	−0.061^**^(0.029) 0.94	−0.045 (0.033) 0.96	−0.048 (0.033) 0.95	0.03 (0.049) 1.03	−0.043 (0.028) 0.96
Housing during the semester: Home (ref.: Away from home)	**−0.152**^†^ **(0.045) 0.86**	**−0.123**^†^ **(0.03) 0.88**	−0.051^*^ (0.028) 0.95	<0.001 (0.032) 1	0.005 (0.032) 1.01	−0.125^***^(0.048) 0.88	−0.087^***^(0.027) 0.92
Form of study: Part-time (ref.: Full-time)	0.042 (0.062) 1.04	−0.016 (0.041) 0.98	−0.012 (0.039) 0.99	0.032 (0.044) 1.03	−0.032 (0.044) 0.97	0.119 ^*^ (0.065) 1.13	0.01 (0.038) 1.01
PHQ-9	**0.171**^†^ **(0.029) 1.19**	**0.16**^†^ **(0.019) 1.17**	**0.125**^†^ **(0.018) 1.13**	**0.069**^†^ **(0.021) 1.07**	**0.135**^†^ **(0.02) 1.14**	**0.122**^†^ **(0.031) 1.13**	**0.14**^†^ **(0.018) 1.15**
PSS-10	**0.209**^†^ **(0.05) 1.23**	**0.123**^†^ **(0.033) 1.13**	**0.121**^†^ **(0.031) 1.13**	0.083^**^(0.035) 1.09	**0.149**^†^ **(0.035) 1.16**	−0.034 (0.051) 0.97	**0.136**^†^ **(0.03) 1.15**
GAD-7	−0.005 (0.037) 0.99	−0.035 (0.024) 0.97	−0.039^*^ (0.023) 0.96	0.011 (0.026) 1.01	−0.053^**^(0.026) 0.95	0.017 (0.039) 1.02	−0.021 (0.023) 0.98
Pseudo *R*^2^ - Nagelkerke	0.139	0.175	0.183	0.056	0.165	0.092	0.192

Significance: ^*^p-value < 0.1,

** *p-value < 0.05*,

*** *p-value < 0.01*,

† *p-value < 0.001*.

*Significant results are highlighted in bold*.

*OR, odds ratio; CZ, Czech Republic; SK, Slovakia; PHQ-9, Patient Health Questionnaire for depressive symptoms; PSS-10, Perceived Stress Scale; GAD-7, Generalized Anxiety Disorder instrument; IAT, Internet Addiction Test*.

Focusing on Slovakia, significant relationships were most frequently found for gender, housing during the semester, PHQ-9 and PSS-10. In the Czech Republic, the results were similar to those in Slovakia, but more significant relationships were found also for age. In both countries, the results showed that male students were more likely to be seriously addicted to the internet compared to female students. Age was particularly significant in the Czech Republic, and the results revealed that younger students were more prone to internet addiction than older ones. Whether a student lived in an urban or rural area was relevant to internet addiction in only one case in both countries. In Slovakia, a slightly higher likelihood of Excessive Use [odds ratio (OR): 1.05] was identified for students living in urban areas. In the Czech Republic, there was a slightly higher likelihood of Lack of Control (OR: 0.93) for students living in rural areas. The family structure (complete/incomplete) in which the students grew up was not so important for internet addiction, as the only significant relationship was found in the Czech Republic. In this case, a slightly higher likelihood of Neglect Work (OR: 0.94) was identified for students from complete families. In terms of housing during the semester, the results indicated that Czech and Slovak students who lived away from home (dormitory, private accommodation, etc.) were more likely to suffer from internet addiction, including several conditions represented by the IAT subscales. The form of study proved to be significant in only one case in Slovakia. More specifically, Slovak full-time students were more likely to report Lack of Control (OR: 0.86) than part-time students. In both countries, depressive symptoms and stress could be considered significant predictors of internet addiction overall, but also in most subscales. Anxiety symptoms were significant in two cases in the Slovak sample. There was a slightly higher likelihood of lower anxiety symptoms and a higher rate of Neglect Work (OR: 0.93) and a slightly higher likelihood of lower anxiety symptoms and a higher rate of Lack of Control (OR: 0.95). One significant relationship was found in the Czech sample. It was possible to observe a slightly higher likelihood of lower anxiety symptoms and a higher rate of Lack of Control (OR: 0.95).

## Discussion

College students are definitely a vulnerable group in terms of psychological distress. They face many challenges during their studies, and therefore the college environment should be adapted to maintain and improve their mental health. Moreover, today's students face another threatening factor, i.e., the COVID-19 pandemic, which has brought with it many concerns and changes in the educational process (66, 67). Evidence shows that COVID-19-related fear (to die, to get sick) was relatively more common among young people (68). They were also worried the influence of COVID-19 on daily life, academic delays due to COVID-19, decreased social support during COVID-19 (69, 70). Thus, given that the pandemic poses a risk to the mental health of college students, it should be given adequate attention in research and practice.

### Prevalence of Internet Addiction

From an overall perspective, during the first wave of the COVID-19 pandemic in the Czech Republic and Slovakia, mild and more severe symptoms of Internet addiction were identified in one third of college students. Moderate and severe symptoms (the cut-off point for IAT was ≥ 50) occurred in 6.02% of Slovak students and 3.5% of Czech students. In an international comparison, Thai students reported a similar prevalence, i.e., 5.8% (71), while substantially higher prevalence rates were observed in other studies. More specifically, 12.4% of internet-addicted students were found in Spain (72), 16.8% in the United States (73), 20% in Brazil (13), 28.4% in China (74), and 32.6% in Bangladesh (75). On this basis, it can be concluded that Czech and Slovak college students did not report such a high prevalence as in other countries, despite the COVID-19 pandemic. A possible explanation is the cultural differences emphasized in the study conducted by Lozano-Blasco et al. (76). However, the problem is mainly the fact that internet addiction is currently not given sufficient attention in the Czech Republic and Slovakia, which may result in serious consequences in the near future.

### Differences and Predictors of Internet Addiction

When assessing internet addiction overall as well as in its subscales, there were differences between students in almost all cases in Slovakia, while in the Czech Republic the occurrence of differences was not as frequent. In more detail, there were several significant differences between males and females. In the case of age, older students reported lower rates of internet addiction. At the same time, students who lived away from home during the semester were found to have significantly higher rates of internet addiction compared to students who lived at home. In terms of the form of study, it can be noted that higher scores for internet addiction and most of its subscales were observed in full-time students. The results for place of residence (urban and rural) and family structure did not show significant differences. The results showed that there were significant differences in internet addiction between the categories of depressive symptoms, anxiety symptoms, and stress symptoms. This finding can be interpreted in such a way that higher mean IAT scores occurred in students with higher levels of psychological distress. The results of the multiple negative binomial regression analysis showed that the main predictors of internet addiction in Czech and Slovak students include gender, age, housing during the semester, depressive symptoms, and stress. The main findings are discussed below.

#### Gender

In both countries, male students were more likely to suffer from internet addiction than female students. Other studies also revealed a higher risk of problematic internet use or internet addiction in male students compared to female students (18, 38, 40–43). The predominance of males in this type of problematic behavior is evident (77). Online behavior is considered more “normal” for males than females. In this context, social norms, socio-cultural attitudes, but also a lower digital participation of females may play an important role in terms of protective factors (78). However, there is also evidence of female susceptibility to internet addiction (79). A possible explanation for this is the pandemic itself, which may have erased male dominance in terms of internet addiction (6). Nevertheless, such a result occurs less frequently in studies. According to Lin et al. (36), the main online activity among college students with moderate and severe internet addiction was online gaming for males and online streaming for females. Having roommates who engage in similar internet entertainment was a risk factor for internet addiction only for males, while the absence of a romantic relationship was a risk factor for internet addiction only for females. Internet infatuation before college and difficulty adjusting to college life were common risk factors for both genders in the groups of students with mild and moderate internet addiction. In any case, gender is a significant factor in the issue of internet addiction (32).

#### Age

In terms of other demographic characteristics, Khazaie et al. (37) revealed that age is also one of the significant predictors of internet addiction. In this study, younger age appeared to be a risk factor especially in the Czech Republic. Similar findings were revealed by Fernández-Villa et al. (12). This is consistent with the findings of Arzani-Birgani et al. (38), who concluded that age is inversely related to internet addiction. Regarding the new younger generations, their greater susceptibility can be explained by an increase of individualism, lower sociability and enculturation (76).

#### Residence

With a focus on place of residence, Slovak urban students showed a slightly higher likelihood of Excessive Use, while Czech rural students showed a slightly higher likelihood of Lack of Control. The identified discrepancy between the analyzed countries requires further investigation. The results can be compared to those of Zewde et al. (40) who found in their study that students from urban areas are more likely to be addicted to the internet. It is generally known that urban areas are more internet accessible and urban individuals make greater use of the internet, which may explain the higher rates of addictive behavior in urban areas (80–82).

#### Housing During the Semester

This study also showed consistent results for housing during the semester. More specifically, Czech and Slovak students who lived away from home (dormitory, private accommodation, etc.) were more likely to be addicted to the internet compared to students who lived at home. The results of Ramón-Arbués et al. (39) also showed that the likelihood of problematic internet use was significantly related to the habitation status of college students. However, the results of this study are not consistent with those of Kivrak and Kivrak (83), who found that dormitory students suffer from internet addiction less than those staying at home. Thus, dormitory housing did not prove to be a protective factor in this study, although dormitory students seem to have more difficult access to the internet.

#### Family

In this study, family structure (complete/incomplete) was not found to be significant in the majority of cases; only one significant relationship with Neglect Work was found in the Czech Republic. This is inconsistent with other studies that emphasize the role of the family environment in the issue of Internet addiction (84, 85). Regarding the form of study, Slovak full-time students were more likely to report Lack of Control compared to part-time students. A similar result was found in a neighboring country, Poland. Thus, increased problematic internet use was observed in full-time students (43). However, this study did not show any other significant relationship between the form of study and internet addiction or its subscale. Thus, family and study form did not play as important a role as in other studies. Possible explanations are cultural differences and different study conditions across countries.

#### Depressive Symptoms, Anxiety Symptoms, Stress

In addition to the socio-demographic profile, significant relationships were also clearly evident for the psychological profile of the students. In this context, Czech and Slovak students with depressive symptoms were more prone to internet addiction. In comparison with national studies, it was also revealed in Slovakia by Sebena et al. (44) that depressive symptoms predict problematic internet use. In international comparison, similar results were confirmed by Yoo et al. (86) and Chi et al. (84). Truzoli et al. (18) also revealed similar results and confirmed that students with severe depression are at higher risk of problematic internet use during the COVID-19 pandemic, while social support, self-efficacy, and self-esteem appeared to be protective factors in their study. It was possible to agree with Werner et al. (6), who found that depressive symptoms were associated with internet addiction in college students, and this phenomenon was observed in both the pre-pandemic and pandemic periods. However, a bidirectional association can also be considered. The fact is that baseline depression has a significant net-predictive effect on follow-up internet addiction, and baseline internet addiction has a significant net-predictive effect on follow-up depression (87). The issue is thus more complex than it seems.

In terms of stress, the results of this study are consistent with those of Yang et al. (31), who found that stress is associated with internet addiction in college medical students. Gong et al. (33) also confirmed a significant predictive effect of perceived stress on internet addiction among college students. Thus, it can be concluded that perceived stress increases the risk of internet addiction among young people (86).

Overall, the findings of this study are consistent with those of Mota et al. (16), who stated that common mental disorders are the variables that best explain problematic internet use, and emphasized that high levels of digital socialization should be considered in mental health care interventions targeting college students in the context of COVID-19. In other words, mental distress is a significant risk factor for internet addiction (88), and the presented study contributes to this knowledge. For students with depression and stress, internet use is believed to be a coping strategy for negative emotions (89–91) or stressful situations (89, 92).

Finally, this study revealed that lower anxiety occurred in students with a higher rate of internet addiction in subscales such as Neglect Work and Lack of Control. These findings can be explained by the fact that higher score on internet addiction may be a protective factor against anxiety in students, which was revealed in a study by Ismail et al. (93). On the other hand, there are inconsistent findings (94, 95). Cai et al. (30) found that students with more severe anxiety symptoms were independently and significantly associated with more severe problematic internet use. In other words, students with a higher level of anxiety tend to have a greater likelihood of internet addiction (32). Thus, it was possible to observe inconsistencies in the results with other studies (6), suggesting that the relationship between anxiety and internet use is a complex issue, as in the case of depression.

Based on all the above-mentioned findings, it was possible to agree that the co-existence of internet addiction and depressive symptoms, anxiety symptoms, and stress in college students indicates that internet addiction is associated with other psychological problems (38). From the opposite point of view, students who were psychologically well were less likely to be addicted (35, 42).

### Implications for Mental Health Policies

Internet addiction and psychological distress in college students are two problems that require increased attention at all times, especially in a difficult period such as a pandemic. The findings of this study may emphasize the importance of the issue and encourage the development of early preventive and protective interventions against problematic internet use to promote the health of college students in the Czech Republic and Slovakia as two countries that are largely neglected in terms of proactive steps. In addition to promoting healthy use of the internet as such, policy makers should take actions to alleviate psychological distress among college students, which could be beneficial in reducing internet addiction. It is recommended to provide college students with the necessary psychological care, especially for symptoms of depression and stress. In these cases, excessive use of the internet is not recommended to improve mood, as it can contribute to deteriorating mental health (17). On the contrary, it is highly recommended to promote the benefits of physical activity (32, 96). This study also suggests that strategies and programs should particularly target male students and students who lived away from home during the semester. All relevant actors, including college teachers, experts, parents and policy makers, should be involved in addressing this problem, which requires a comprehensive approach. This is emphasized particularly in countries such as the Czech Republic and Slovakia, where it is evident that insufficient attention is being paid to this problem, which may have more serious consequences in the future. In these countries, access to help for students should be improved, both in the college environment and in the specialized counseling centers. In this context, it is recommended to identify in time the students who need this help, as well as to provide relevant assistance to students seeking help. However, all these steps inevitably require social, legislative and professional support, which is currently underestimated and insufficient in both countries (97). The authors call for greater research interest in this issue and for the implementation of evidence-based measures into practice in the Czech and Slovak conditions.

### Strengths and Limitations

This study has many strengths, which include in particular the sample size, but also the uniqueness of the findings for mental health policies in the Czech Republic and Slovakia, where the issue is under-researched and poorly evidence-based. The findings of this study fill a substantial research gap in this region and can therefore be considered novel. The study offers a detailed statistical insight into the issue and provides a valuable basis for further research, as well as effective mechanisms and successful strategies and programs.

The study did not avoid certain limitations. In this respect, the situation in the Czech Republic and Slovakia during the pandemic did not allow for a fully balanced sample. For this reason, the ratio of male to female students was slightly skewed in favor of female students (especially in the Czech Republic). As males are generally more addicted to the internet compared to females, there may have been some bias. Differences in the duration and mode of online (distance) learning in the Czech Republic and Slovakia may have had some, but minimal, effects on the sample. During the pandemic, distance learning was implemented to a greater extent in Slovakia than in the Czech Republic. In terms of country development, it can be assumed that the initial implementation of distance learning in Slovakia was of lower quality than in the Czech Republic. However, the authors do not expect this bias to be so significant as to substantially affect the results.

The research was conducted during the pandemic characterized by many specifics (online learning, isolation, social distance, etc.); therefore, future research should focus on the post-pandemic state.

## Conclusion

Internet addiction among young adults is a phenomenon of recent years that has been more highlighted by the COVID-19 pandemic. Physical distance has required college students to use the internet for virtually all daily activities during the pandemic. For this reason, it is important to identify and monitor vulnerable groups in order to prevent the situation from worsening in the future. However, this cannot be done without knowledge of the risk factors that commonly characterize students and which should be the focus of proactive actions. All of this was the motivation for conducting the presented study, which aimed to assess the relationships between internet addiction and selected socio-demographic, study-related, and psychological characteristics of college students in the Czech Republic and Slovakia. In addition, the rationale for the research was based on the previous lack of attention in these two countries. This study mapped the situation and pointed to a problem that could have serious consequences in the future. The main findings showed that male students and students who lived away from home during the semester were more likely to be addicted to the internet both overall and in terms of most of the individual subscales of internet addiction. Depressive symptoms and stress could also be considered significant predictors in both countries. Thus, these groups of students can be identified as vulnerable and should be targeted by protective and preventive actions. The existing relationships between internet addiction and positive screening for psychological distress and other demographic and academic characteristics of students are an alert for professionals to address and further investigate this serious issue. Knowledge of these relationships provides an opportunity to implement effective protective and preventive measures, such as psychoeducation, and to offer appropriate treatment.

## Data Availability Statement

The original contributions presented in the study are included in the article/supplementary material, further inquiries can be directed to the corresponding author.

## Ethics Statement

The research was approved by the Ethics Committee of the General University Hospital in Prague as individual research (Ref. 915/20 S–IV). The study was conducted according to the guidelines of the Declaration of Helsinki. All respondents were over 18 years of age and provided their informed consent to participate in this research.

## Author Contributions

BG: conceptualization, investigation, writing—original draft preparation, writing—review and editing, visualization, supervision, project administration, and funding acquisition. VI: conceptualization, methodology, investigation, resources, writing—original draft preparation, writing—review and editing, visualization, supervision, and funding acquisition. MR: conceptualization, methodology, software, data curation, formal analysis, investigation, writing—original draft preparation, writing—review and editing, and visualization. TM: conceptualization, investigation, resources, writing—original draft preparation, writing—review and editing, visualization, supervision, and project administration. All authors contributed to manuscript revision, read, and approved the submitted version.

## Funding

This research was funded by the Scientific Grant Agency of the Ministry of Education, Science, Research, and Sport of the Slovak Republic and the Slovak Academy Sciences as part of the research project VEGA 1/0797/20: Quantification of Environmental Burden Impacts of the Slovak Regions on Health, Social and Economic System of the Slovak Republic. This research was supported by the Slovak Research and Development Agency under the contract No. APVV-17-0360: Multidimensional analysis of significant determinants of public procurement efficiency with emphasis on the application of Health Technology Assessment in the procurement preparation phase. This study was supported by Institutional Research Program PROGRES No. Q06/LF1.

## Conflict of Interest

The authors declare that the research was conducted in the absence of any commercial or financial relationships that could be construed as a potential conflict of interest.

## Publisher's Note

All claims expressed in this article are solely those of the authors and do not necessarily represent those of their affiliated organizations, or those of the publisher, the editors and the reviewers. Any product that may be evaluated in this article, or claim that may be made by its manufacturer, is not guaranteed or endorsed by the publisher.
